# Endogenous Neural Stem Cell Activation after Low-Intensity Focused Ultrasound-Induced Blood–Brain Barrier Modulation

**DOI:** 10.3390/ijms24065712

**Published:** 2023-03-16

**Authors:** Younghee Seo, Sangheon Han, Byeong-Wook Song, Jin Woo Chang, Young Cheol Na, Won Seok Chang

**Affiliations:** 1 Department of Neurosurgery and Brain Research Institute, Yonsei University College of Medicine, Seodaemun-gu, Seoul 03722, Republic of Korea; 2 Brain Korea 21 Project for Medical Science, Yonsei University College of Medicine, Seodaemun-gu, Seoul 03722, Republic of Korea; 3 Department for Medical Science, College of Medicine, Catholic Kwandong University, Gangwon-do, Gangneung City 25601, Republic of Korea; 4 Department of Neurosurgery, Catholic Kwandong University College of Medicine, International St. Mary’s Hospital, Seo-gu, Incheon Metropolitan City 22711, Republic of Korea

**Keywords:** low-intensity focused ultrasound, endogenous neural stem cells, neurogenesis, fluoro-L-thymidine, Sox-2, nestin

## Abstract

Endogenous neural stem cells (eNSCs) in the adult brain, which have the potential to self-renew and differentiate into functional, tissue-appropriate cell types, have raised new expectations for neurological disease therapy. Low-intensity focused ultrasound (LIFUS)-induced blood–brain barrier modulation has been reported to promote neurogenesis. Although these studies have reported improved behavioral performance and enhanced expression of brain biomarkers after LIFUS, indicating increased neurogenesis, the precise mechanism remains unclear. In this study, we evaluated eNSC activation as a mechanism for neurogenesis after LIFUS-induced blood–brain barrier modulation. We evaluated the specific eNSC markers, Sox-2 and nestin, to confirm the activation of eNSCs. We also performed 3′-deoxy-3′[^18^F] fluoro-L-thymidine positron emission tomography ([^18^F] FLT-PET) to evaluate the activation of eNSCs. The expression of Sox-2 and nestin was significantly upregulated 1 week after LIFUS. After 1 week, the upregulated expression decreased sequentially; after 4 weeks, the upregulated expression returned to that of the control group. [^18^F] FLT-PET images also showed higher stem cell activity after 1 week. The results of this study indicated that LIFUS could activate eNSCs and induce adult neurogenesis. These results show that LIFUS may be useful as an effective treatment for patients with neurological damage or neurological disorders in clinical settings.

## 1. Introduction

The discovery of endogenous neural stem cells (eNSCs) in the adult brain, which have the potential to self-renew and specialize into tissue-appropriate functional cell types, has raised new expectations for neurological disease therapy [1]. These rare, slowly dividing cells are present throughout the neuraxis of the developing and mature central nervous system (CNS). eNSCs persist in the brains of patients with neurodegenerative disorders, albeit at much lower densities [2]. The subgranular zone (SGZ) of the dentate gyrus in the hippocampus and the subventricular zone (SVZ) of the lateral ventricles generate eNSCs in the adult brain [3]. Recent studies have reported that adding new neurons into the existing hippocampal circuitry, known as adult hippocampal neurogenesis, persists throughout aging, although it drops sharply in patients with Alzheimer’s disease [4,5]. The adult organ retains stem cells and can constantly produce new cells or perform this function in response to injury. Indeed, eNSCs, which can develop into neurons, astrocytes, and oligodendrocytes, are still present in the adult brain and spinal cord [6,7,8]. Continuous neurogenesis occurs in the brain because of eNSCs with persistent pluripotency, multipotency, and plasticity [9].

Low-intensity focused ultrasound (LIFUS) combines with microbubbles to generate stable cavitation and can modulate the blood–brain barrier (BBB) [10]. Low frequencies are mainly used because the distortion and attenuation are lesser than those for high frequencies [11]. Therefore, this is a promising drug delivery method across the BBB to the CNS [12].

This technology has also been reported to regulate immune responses, improve cognitive function, and promote neurogenesis [13,14]. Although several studies have shown improved performance on behavioral tests and enhanced brain biomarker expression, indicating increased neurogenesis after LIFUS, the precise process underlying this phenomenon remains unclear.

Therefore, in this study, we evaluated eNSC activation as a mechanism of neurogenesis after LIFUS-induced BBB modulation. We evaluated the eNSC markers, Sox-2 and nestin, to confirm the activation of eNSCs. We also performed 3′-deoxy-3′[^18^F] fluoro-L-thymidine positron emission tomography ([^18^F] FLT-PET) to evaluate the activation of eNSCs in living animals.

## 2. Results

### 2.1. Low-Intensity Focused Ultrasound-Induced Blood–Brain Barrier Modulation

**Figure 1:**
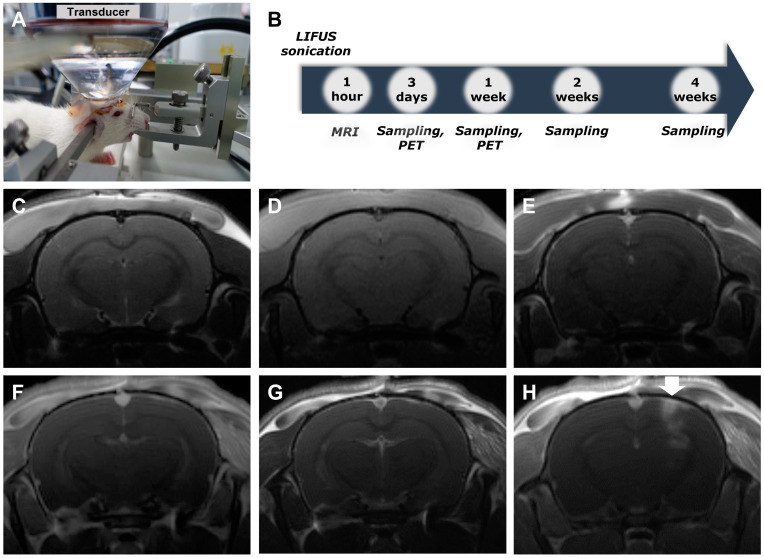
Confirmation of LIFUS-induced BBB modulation by MRI imaging. (**A**) FUS experimental system setup. (**B**) Timeline of the experiment for comparison at 3 days, 1 week, 2 weeks, and 4 weeks after sonication. (**C**) T2-weighted image of non-treated rats. (**D**) T1-weighted image of non-treated rats. (**E**) Gadolinium-enhanced T1-weighted image of non-treated rats. (**F**) T2-weighted image of treated rats. (**G**) T1-weighted image of treated rats. (**H**) Gadolinium-enhanced T1-weighted image of treated rats. Arrow: modulated area of LIFUS. BBB, blood–brain barrier; LIFUS, low-intensity focused ultrasound; MRI, magnetic resonance imaging; PET, positron emission tomography.

After LIFUS was performed, targeting the right hippocampus, magnetic resonance imaging (MRI) confirmed that the BBB was safely modulated. On T2-weighted images, it was confirmed that there was no edema caused by LIFUS (Figure 1F). T1-weighted images were obtained without the Dotarem contrast (Figure 1G). The contrast agent was then injected, and T1-enhanced images were obtained 1 min later to confirm that the BBB was modulated by LIFUS (Figure 1H).

### 2.2. Upregulated Endogenous Neural Stem Cell Markers after Low-Intensity Focused Ultrasound-Induced Blood–Brain Barrier Modulation

**Figure 2:**
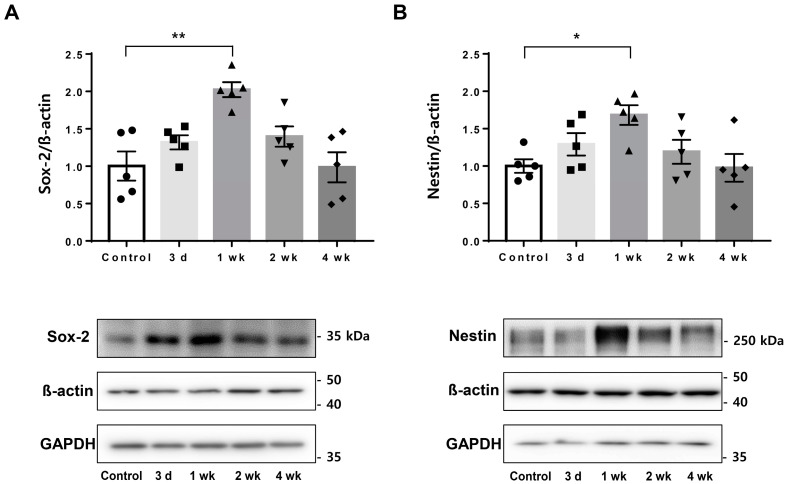
Comparison of eNSC markers by using LIFUS. (**A**) Western blotting for Sox-2 comparing the control and LIFUS groups. (**B**) Western blotting for nestin comparing the control and LIFUS groups. Data are expressed as the mean ± standard error of the mean. *n* = 5 for each group. * *p* < 0.05, ** *p* < 0.01; one-way ANOVA with Tukey’s post hoc comparisons was used to analyze the data. eNSC, endogenous neural stem cell.

Sampling was performed at 3 days, 1 week, 2 weeks, and 4 weeks after treatment of the rat hippocampus with LIFUS, and PET scans were performed at 3 days and 1 week. The endogenous neural stem cell markers, Sox-2 and nestin, were detected via Western blotting. It was confirmed that the levels of both markers increased from the third day after LIFUS, and the largest increase was observed in the first week, indicating significance. The increased pattern was maintained in the second week and returned to the control state in the fourth week (Figure 2).

### 2.3. Co-Expression of Endogenous Neural Stem Cell Markers

**Figure 3:**
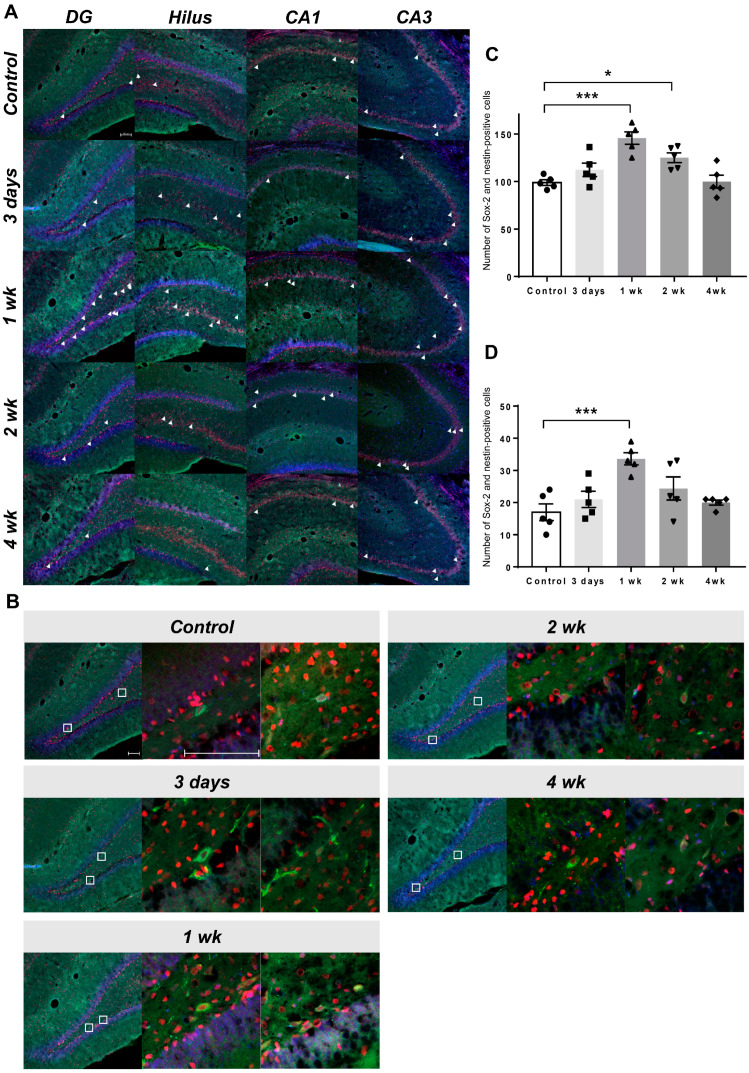
Co-immunostaining for Sox-2 and nestin in control and LIFUS groups. (**A**) Histological staining with DAPI (blue), anti-Sox-2 (red), and anti-nestin (green) in the DG, hilus, CA1, and CA3 of the hippocampus. White arrow: Sox-2 and nestin-positive cells. (**B**) 10x photo of the DG of each group and 40x magnification of the co-expression region. White square: enlarged region. (**C**) Results of co-localization of Sox-2 and nestin-positive cells in hippocampus. (**D**) Results of co-localization of Sox-2 and nestin-positive cells in DG. Data are expressed as the mean ± standard error of the mean. *n* = 5 animals for each group. * *p* < 0.05, *** *p* < 0.001; one-way ANOVA with Tukey’s post hoc comparisons was used to analyze the data. Scale bar, 100 μm. CA, cornu ammonis; DAPI, 4′,6-diamidino-2-phenylindole; DG, dentate gyrus.

Co-immunostaining for Sox-2 and nestin confirmed that the cells were eNSCs (Figure 3A,C). It was determined that the increase started from the third day after LIFUS, was the highest at 1 week, and became similar to the control level at the fourth week. This was determined by counting the co-expressing cells and confirming each group (Figure 3B). 

### 2.4. Visualization of Upregulated Endogenous Neural Stem Cell Activation Using [^18^F] Fluoro-L-Thymidine Positron Emission Tomography

**Figure 4:**
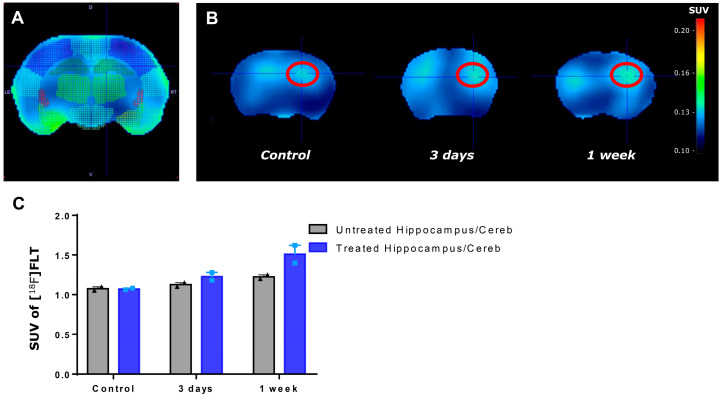
[^18^F] FLT activation by using LIFUS. (**A**) Identification of the site using the rat brain atlas. (**B**) [^18^F] FLT-PET was taken at 3 days and 1 week in the control and LIFUS groups. (**C**) Comparison of SUV between the untreated and treated hippocampus in each group. Cereb—cerebellum; FLT—fluoro-L-thymidine; SUV—standardized uptake value.

[^18^F] FLT-PET was performed 3 days and 1 week after LIFUS. PET images were categorized into regions using the rat atlas of PMOD (Figure 4A). It was confirmed that more tracer was detected in the right hippocampus of rats treated with LIFUS (Figure 4B). When verifying the standardized uptake value by dividing the values of the treated hippocampus and the untreated hippocampus by the reference cerebellum, the values of the LIFUS group increased as a whole, and the values of the treated area were the highest in the first week (Figure 4C).

## 3. Discussion

### 3.1. Low-Intensity Focused Ultrasound-Induced Blood–brain Barrier Modulation

Depending on the intensity, focused ultrasound is largely classified into high-intensity focused ultrasound (HIFU) and LIFUS. HIFU produces temperatures high enough to denature proteins and coagulate tissue and is often used to remove fibroids, cancers, or skull tumors [15,16].

Compared with HIFU, LIFUS can temporarily and reversibly modulate the BBB when combined with microbubbles [17,18]. Previously, we reported the optimal parameters for improving BBB permeability using LIFUS [19] and confirmed the improvement of cognitive function by neurogenesis [20]. Another report confirmed the therapeutic effect in an Alzheimer’s disease animal model by increasing the drug delivery effect through BBB modulation [12]; another previous study improved the delivery rate with mesenchymal stem cells [21].

In addition, LIFUS can promote the differentiation of pluripotent stem cells and neurogenesis [20,22,23]. A few studies reported that only LIFUS conditions sufficient to induce and modulate increased BBB permeability could promote neurogenesis [24]. Therefore, in this study, we selected a parameter capable of BBB modulation via LIFUS to evaluate eNSC activation.

### 3.2. Endogenous Neural Stem Cell-Induced Neurogenesis

In the brain, new neurons are produced in the SVZ around the ventricle and the SGZ in the hippocampus, where neurogenesis occurs most actively and continuously [25,26]. Many studies have shown that when the brain is abnormal, the dividing neuroblasts move to the lesion location, and the migrated cells surround the lesion and slow its progression [27,28,29].

As such, there have been attempts to treat brain lesions by activating endogenous neurogenesis. However, the neuroblasts that divide and migrate toward the lesion undergo a process of cell annihilation rapidly over time [30]. Accordingly, attempts have been made to ensure the long-term survival of neuroblasts by injecting neurotrophic factors, such as vascular endothelial growth factor, epidermal growth factor, and brain-derived neurotrophic factor, into the brain, which increase the differentiation and survival of eNSCs originally present in vivo [31,32].

### 3.3. Endogenous Neural Stem Cell Activation after Low-Intensity Focused Ultrasound-Induced Blood–Brain Barrier Modulation

Recently, many studies reported the effectiveness of LIFUS for drug delivery and BBB modulation [21,33,34]. In addition, many studies report neurogenesis using the thymidine analog 5-bromo-2′-deoxyuridine (BrdU) increase, an established immunodetection method used to identify proliferating cells after LIFUS-induced BBB modulation [20,24].

We evaluated eNSC markers Sox-2 and nestin to confirm enhanced neurogenesis after LIFUS-induced BBB modulation based on these previous results. Tissue staining identified the upregulated expression of Sox-2 (neural progenitor cell marker) and nestin (immature neuron marker), and a co-expression increase was observed. In addition, the morphology of eNSCs was confirmed by DAB staining (Supplementary Figure S1).

It has been reported that an increase in Sox-2 and nestin double-positive cells shows the possibility of neurogenesis by neural precursor cells [35,36]. Additionally, since there are reports that Sox-2 and nestin are related to reactive astrocytes [37], they were co-stained with glial fibrillary acidic protein (GFAP); it was then confirmed that Sox-2 was partially overlapped with astrocytes, and nestin was not overlapped with astrocytes (Supplementary Figures S2 and S3). In this study, we also observed upregulated activation of eNSCs because of the increase in Sox-2 and nestin double-positive cells after LIFUS-induced BBB modulation.

A few studies reported that PET using 3′-deoxy-3′[^18^F] fluoro-L-thymidine ([^18^F] FLT) enables the imaging and measurement of eNSC proliferation [1]. However, this was shown as a new way to overcome many limitations, which had to be evaluated by sacrificing experimental animals and using immunohistochemical staining to evaluate eNSCs in an in vivo environment. We identified eNSCs after LIFUS-induced BBB modulation in live animals using the capability of FLT-PET.

In summary, we observed the upregulated expression of Sox-2 and nestin, and high uptake in [^18^F] FLT-PET imaging, which indicate eNSC activation. Nonetheless, in this study, the detailed activation and reduction of eNSCs could not be confirmed after LIFUS-induced BBB modulation. Moreover, we could not confirm the results of repeated LIFUS treatment at the time when eNSC activation decreased through FLT-PET. Furthermore, follow-up studies on the activation of eNSCs by LIFUS in various brain diseases and the mechanisms of cell differentiation are needed.

## 4. Materials and Methods

### 4.1. Animals

All animal experiments were performed according to the *Guide for the Care and Use of Laboratory Animals* of the National Institutes of Health, and approved by the Institutional Animal Care and Use Committee of Yonsei University (South Korea) (IACUC number: 2022-0068). Male Sprague Dawley rats (*n* = 56, 260–300 g) were categorized into a control group (*n* = 12), which received no treatment, and four LIFUS groups, which were sacrificed 3 days (*n* = 12), 1 week (*n* = 12), 2 weeks (*n* = 10), and 4 weeks (*n* = 10) following LIFUS sonication for BBB modulation.

### 4.2. Low-Intensity Focused Ultrasound-Induced Blood–Brain Barrier Modulation

Ketamine (75 mg/kg), acepromazine (0.75 mg/kg), and xylazine (4 mg/kg) were used to anesthetize the animals. The animals were then fixed to a stereotaxic frame using ear and nose bars. After the skin was incised, the cone was fixed to the skull. The LIFUS apparatus consists of a 515 kHz single-element spherically focused H-107MR transducer (Sonic Concept Inc., Bothell, WA, USA), a waveform generator (33220A; Agilent, Palo Alto, CA, USA), and a radiofrequency power amplifier (240 L; ENI Inc., Rochester, NY, USA). LIFUS parameters were determined according to a previous study [19]. The cone was positioned over the right hippocampus (anteroposterior −3.5 mm; mediolateral +2.5 mm from the bregma), the LIFUS targeting site, and DEFINITY^®^ microbubbles (mean diameter range, 1.1–3.3 µm) (Lantheus Medical Imaging, North Billerica, MA, USA) were injected through the tail vein. The average peak-negative pressure was set at 0.25 MPa by using a burst duration of 10 ms and pulse repetition frequency of 1 Hz over 120 s.

MRI was performed using a rat head coil and 9.4-T 20 cm bore-diameter MRI system (BioSpec 94/20 USR; Bruker, Ettlingen, Germany) one hour after sonication. After obtaining T2- and T1-weighted images, Dotarem (gadoterate meglumine; Guerbet, Villepinte, France), a gadolinium-based contrast agent, was injected. Subsequently, contrast-enhanced T1-weighted images were acquired to confirm LIFUS-mediated BBB modulation.

### 4.3. Immunohistochemistry

After LIFUS sonication, five animals in each group were sacrificed and perfused with 0.9% saline and 4% paraformaldehyde. The brains were acquired and sectioned into 30 µm slices using a microtome (Leica Biosystems, Wetzlar, Germany). The slices were placed in a cryoprotectant solution of 0.1 M phosphate buffer (pH 7.2)—30% sucrose, 1% polyvinylpyrrolidone (Sigma-Aldrich, St. Louis, MO, USA), and 30% ethylene glycol (Thermo Fisher Scientific, Rockford, IL, USA)—and stored at −20 °C.

Brain tissues were subjected to antigen retrieval in 2 N HCl for 1 h and neutralized twice with 0.1 M borate buffer for 10 min to identify Sox-2 and nestin. The tissues were blocked with 5% normal goat serum for 1 h after washing with phosphate-buffered saline (PBS). Primary antibodies against Sox-2 (1:250, ab97959, Abcam, Cambridge, UK) and nestin (1:250, GTX630201, GeneTex, Irvine, CA, USA), diluted in PBS containing 0.3% Triton X-100 (Sigma-Aldrich), were applied to the tissues and incubated overnight at 4 °C, followed by incubation with secondary antibodies conjugated with Alexa Fluor 633 (A21071, 1:500, Thermo Fisher Scientific) or Alexa Fluor 488 (A11001, 1:500, Thermo Fisher Scientific).

Analyses of Sox-2 and nestin co-localization were performed in the dentate gyrus, hilus, and cornu ammonis (CA1 and CA3). Staining intensity was visualized using an LSM 700 confocal microscope (Carl Zeiss, Jena, Germany).

### 4.4. Western Blot Analysis

The brains were removed, and the right hippocampus (anteroposterior −3.5 mm; mediolateral +2.5 mm from the bregma) was dissected after the animals (*n* = 5 per group) were anesthetized. The tissues were homogenized with lysis buffer (PRO-PREP, catalog no. 17081; iNtRON Biotechnology, Seongnam, Korea) using a pellet pestle (Kimble). The protein concentration was measured using the Pierce Bicinchoninic acid Protein Assay Kit (Thermo Fisher Scientific, Waltham, MA, USA).

Proteins were separated using 12% or 5% sodium dodecyl sulfate-polyacrylamide gels and electrotransferred onto polyvinylidene fluoride membranes to confirm Sox-2 or nestin expression. The membranes were blocked with 5% skim milk (BD Difco) in Tris-buffered saline with Tween (Sigma-Aldrich, St. Louis, MO, USA).

Membranes were then incubated with primary antibodies Sox-2 (SC365823, 1:100, Santa Cruz Biotechnology), nestin (GTX630201, 1:1000, GeneTex), β-actin (A5441, 1:20,000, Sigma-Aldrich), and GAPDH (2118, 1:2000, Cell Signaling Technology), and stored overnight at 4 °C. The secondary antibody, horseradish peroxidase (HRP)-conjugated goat anti-mouse IgG(H+L) or goat anti-rabbit IgG(H+L)-HRP (GenDEPOT, Katy, TX, USA), was applied at 25 °C for 2 h.

The proteins were detected using an enhanced chemiluminescence solution (West Save, Western blot detection kit, Ab frontier). Signals were obtained using Amersham ImageQuant 800 (GE Healthcare Life Sciences, Chicago, IL, USA). In addition, band signals were evaluated using an analytical system (Multi Gauge version 3.0; Fujifilm, Tokyo, Japan).

### 4.5. Positron Emission Tomography and Image Analysis

PET scans were performed in the control group, 3 days and 1 week after LIFUS, and all rats were injected with 2 μCi of [^18^F] FLT through intravenous injection under isoflurane anesthesia. PET scans were acquired for 90 min using a Siemens Inveon scanner (Siemens, Knoxville, TN, USA). Additionally, the images were reconstructed using an ordered subset expectation maximization algorithm with attenuation, scatter, and random correction. The voxel size was 0.776 × 0.776 × 0.796 mm. All reconstructed images were normalized according to the rat brain template (PMOD 4.2, PMOD Technologies Ltd., Zürich, Switzerland).

### 4.6. Statistical Analysis

Data were analyzed using one-way analysis of variance (ANOVA) with Tukey’s post hoc comparisons using GraphPad Prism 7 (GraphPad Software 7, Inc., San Diego, CA, USA). The mean ± standard error of the mean was used to present the data. Statistical significance was set at * *p* < 0.05, ** *p* < 0.01, and *** *p* < 0.001.

## 5. Conclusions

This study evaluated the possibility of eNSC activation after LIFUS-induced BBB modulation. The results of this study demonstrated the ability to visualize the degree of activation after LIFUS treatment and its lasting effects through histology, Western blotting, and PET imaging. LIFUS is expected to be useful as an effective treatment for patients with neurological damage or neurological disorders caused by external factors.

## Data Availability

The data presented in this study are available upon request from the corresponding author.

## Supplementary Materials

The following supporting information can be downloaded at: https://www.mdpi.com/article/10.3390/ijms24065712/s1.
