# Supplementary material for: Endogenous Neural Stem Cell Activation after Low-Intensity Focused Ultrasound-Induced Blood–Brain Barrier Modulation
Source: Int J Mol Sci. 2023 Mar 16;24(6):5712. doi: 10.3390/ijms24065712 (PMC10056062; doi:10.3390/ijms24065712)

Figure S1. DAB staining for (A) Sox-2 (B) nestin in control and LIFUS groups. Scale bar, 100um(20x), 50um(40x)

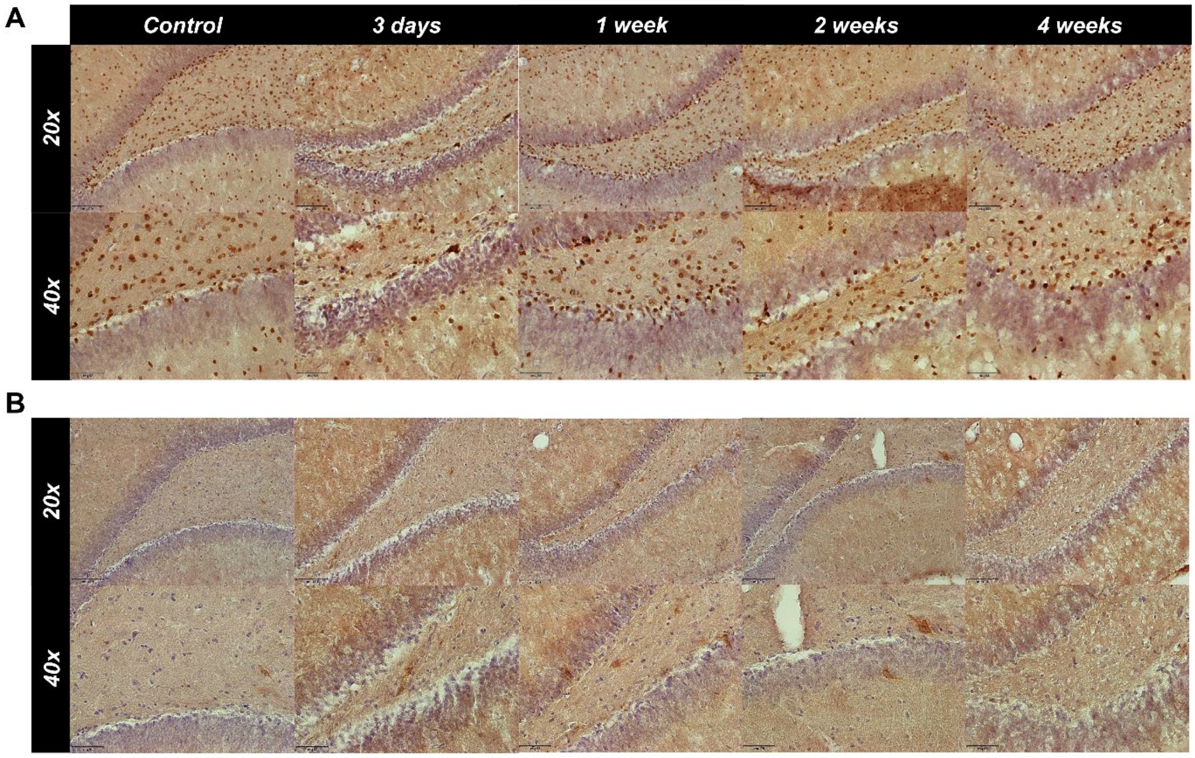

Figure S2. Co-immunostaining for DAPI (blue), anti-Sox-2 (red), and anti-GFAP (green) in control and LIFUS groups. Scale bar, 50 μm

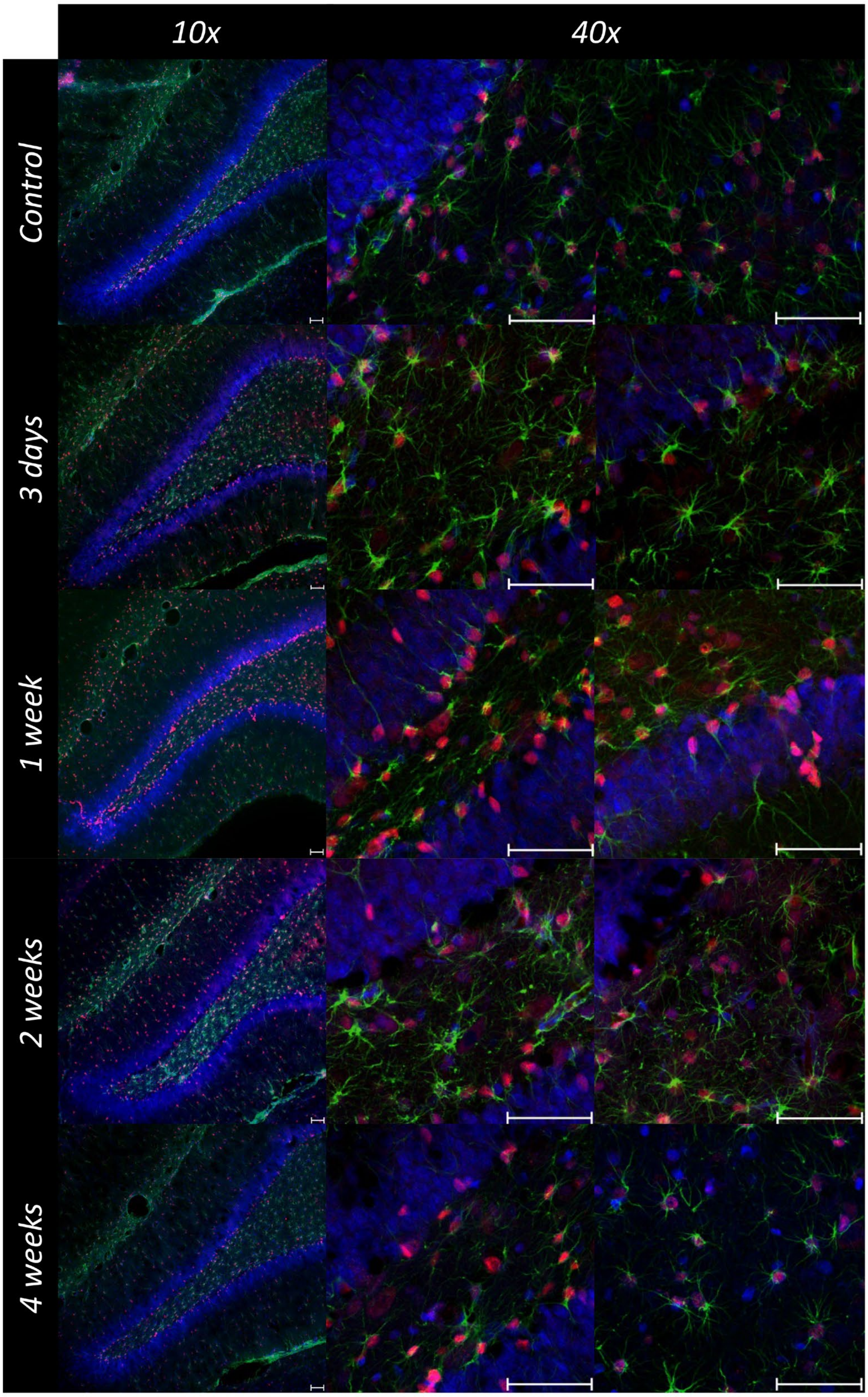

Figure S3. Co-immunostaining for DAPI (blue), anti-nestin (red), and anti-GFAP (green) in control and LIFUS groups. Scale bar, 50 μm

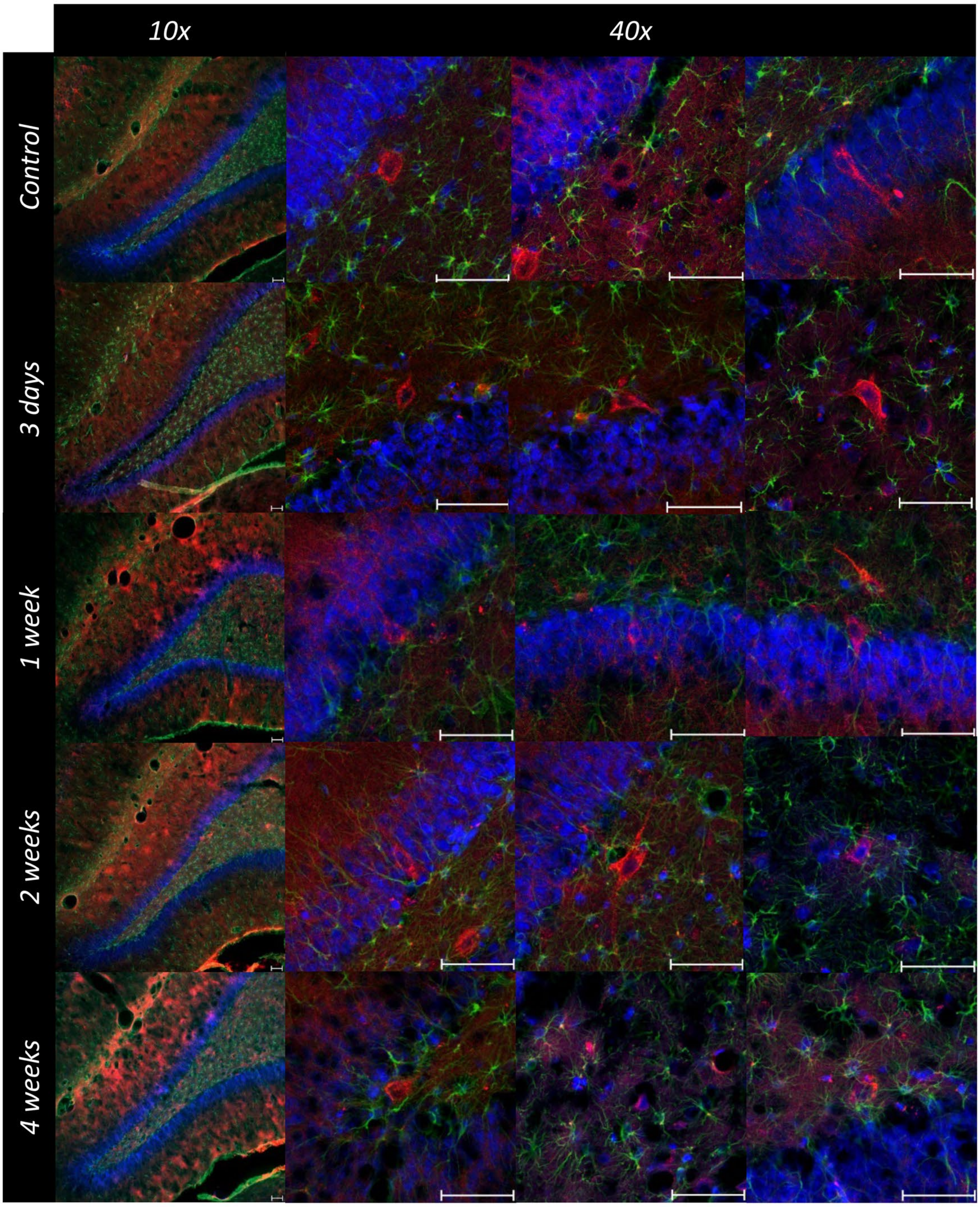

Supplement: Supplementary file 1 [file ijms-24-05712-s001.zip › ijms-2212707-supplementary.pdf]
